# The impact of COVID-19 pandemic on sleep quality in healthcare workers in Turkey

**DOI:** 10.1186/s41983-022-00489-3

**Published:** 2022-05-20

**Authors:** Ayşegül Erdoğan, Deniz Tuncel Berktaş, Ali Nuri Öksüz, Ahmet Rıza Şahin, Burhan Fatih Koçyiğit

**Affiliations:** 1 Faculty of Medicine, Department of Public Health, Kahramanmaraş Sütçü İmam University, Batı Çevreyolu Blv. No:251/A 46040, Onikişubat, Kahramanmaraş, Turkey; 2 Faculty of Medicine, Department of Neurology, Kahramanmaraş Sütçü İmam University, Kahramanmaraş, Turkey; 3 Kahramanmaras Provincial Health Directorate, Kahramanmaraş, Turkey; 4 Faculty of Medicine, Department of Infectious Diseases and Clinical Microbiology, Kahramanmaraş Sütçü İmam University, Kahramanmaraş, Turkey; 5 Department of Physical Medicine and Rehabilitation, Faculty of Medicine, Kahramanmaraş Sütçü İmam University, Kahramanmaraş, Turkey

**Keywords:** COVID-19, Sleep quality, Healthcare workers, Jenkins Sleep Scale

## Abstract

**Background:**

The COVID-19 pandemic has caused serious concerns and psychological distress globally. Healthcare workers remain one of the most affected groups due to life threatening risks in addition to increased working hours and labor intensity. All these factors may affect sleep quality of this population. The aim of this study is to evaluate the sleep behaviors of healthcare professionals working in secondary and tertiary hospitals in a large population in Turkey and to show how sleep quality is affected during the pandemic process using the easily applicable Jenkins Sleep Scale (JSS). The population of this cross-sectional descriptive study consists of two pandemic hospitals determined in Kahramanmaraş province. In our questionnaire, we asked subjective sleep quality, sleep time, time to fall asleep, total sleep time, and medication use. We also used JSS Turkish version (JSS-TR) to assess sleep quality and the Epworth Sleepiness Scale (ESS) for increased daytime sleepiness.

**Results:**

Healthcare workers who participated in our survey reported that they started to go to bed later, fell asleep later (mean: 41.75 ± 35.35 min), their total sleep time (mean: 6.67 ± 1.88 h) was shortened, and they needed medication to sleep more (5.7%) after the COVID-19 pandemic. During the COVID-19 pandemic, bedtime behavior after 24:00 decreased from 80.1 to 43.9% of those who previously went to bed before 24:00. For those who went to bed after 24:00 before, it increased from 19.9 to 56.1%. In addition, sleep quality as assessed by subjective and JSS significantly deteriorated after the COVID-19 pandemic. Excessive daytime sleepiness increased. Those with ESS > 10 before and after COVID-19 were 3.9% and 14.1%, respectively (*p* < 0.001).

**Conclusions:**

The COVID-19 pandemic has significantly adversely affected the sleep behavior and sleep quality of healthcare professionals. The JSS is an easily applicable scale for assessing sleep quality in large population studies.

## Introduction

Coronavirus disease-19 (COVID-19) caused by the severe acute respiratory syndrome coronavirus 2 (SARS COV-2) first emerged in Wuhan towards the end of 2019. The pandemic is now influencing the whole world with a large number of deaths in addition to many other medical, mental and social consequences. While the average population show normal responses to pandemic stressors, others who are more mentally vulnerable to anxiety have more emotional responses. Catastrophic thinking can trigger emotional reactions in the healthy population, such as (1) panic disorder with anxiety, (2) specific phobias, (3) obsessive compulsive disorder, (4) post-traumatic stress disorder (PTSD), and (5) pain [1–3].

Sleep plays a unique role in the maintenance of immunity; the circumstances that affect its quality have been associated with a reduction in the response to vaccines and an increase in vulnerability to infectious diseases. Sleep can be affected in the COVID-19 pandemic, which naturally causes an increase in anxiety and stress, contributing to the deregulation of inflammatory and antiviral responses [4]. There are various studies showing that sleep quality is significantly affected in healthcare workers. The reason for this is that healthcare workers are prone to increased working hours and labor intensity in the face of serious epidemics, not being able to find enough time to rest, chronic stress and psychological distress [5, 6]. The aim of this study was to evaluate the sleep behaviors of healthcare workers working in secondary and tertiary hospitals in a large population in Turkey, and we wanted to show how sleep quality was affected during the pandemic process using the easily applicable Jenkins Sleep Scale (JSS).

## Methods

The population of this cross-sectional descriptive study consists of healthcare workers of two pandemic hospitals (2nd level state hospital and 3rd level university hospital) determined in Kahramanmaraş province. Specialist physicians, assistant physicians and assistant health personnel (nurse, health officer, health technician, laboratory technician, radiology technician, medical secretary) were included in the scope of the research. 106 specialist physicians, 602 assistant health personnel, 237 assistant physicians and approximately 550 assistant health personnel work in the Medical Faculty Hospital in the 2nd step state hospital. In total, the number of physicians is 343 and the number of auxiliary health personnel is 1152. It is aimed to reach at least half of the health workers. Those who did not accept the questionnaire and gave incomplete answers to the questions were not evaluated and 742 participants were included in the study.

Data were collected between 03.08.2020 and 30.09.2020 with responses to online survey questions. The security of the data was assigned to SurveyMonkey enterprise. In our questionnaire, we asked subjective sleep quality (poor, moderate, high), sleep time (< 24:00 and ≥ 24:00), time to fall asleep (minutes), total sleep time (hours), and medication use (sleeping pills). We also used Jenkins Sleep Scale Turkish version (JSS-TR) to assess sleep quality and the Epworth Sleepiness Scale (ESS) for increased daytime sleepiness.

JSS-TR questionnaire consists of four items that assess the sleep problems over the preceding 4 weeks: (a) trouble falling asleep, (b) trouble staying asleep, (c) wake up several times/night, and (d) wake up feeling tired. Each item is rated on a 6-point Likert scale (not at all = 0, 1–3 days = 1, 4–7 days = 2, 8–14 days = 3, 15–21 days = 4, 22–28 days = 5). The total score is ranging from 0 to 20, showing more disturbed sleep as it increases. Duruoz et al. tested the JSS-TR's validity and reliability for Turkey (Cronbach's alpha ≥ 0.86) [7, 8].

The ESS consists of eight items, and it measures a participant's self-reported daytime sleepiness. The instrument focuses on the expectation of “dozing” in a variety of situations. The probability ratings in hypothetical situations are zero (0), slight (1), moderate (2), or high (3). The ratings can be summarized to a total score of 24, with a cutoff value of > 10 indicating excessive daytime sleepiness. Several studies have used the ESS and it is a well-validated questionnaire. Izci and colleagues tested the ESS's validity and reliability for Turkey (Cronbach's alpha ≥ 0.86) [9, 10].

All data were entered and analyzed using Statistical Package for Social Sciences (SPSS) for Windows version 20.0 (IBM Corp., Chicago). Conformity of the data to normal distribution was assessed with the Shapiro–Wilk test and it was determined that the data were not normally distributed. Data were expressed as number (n) and percentage (%) and median (minimum–maximum) values. For the categorical variables, group comparisons were performed using the Chi-Square test. Student t test was used to evaluate the data obtained by measuring in independent groups. In the evaluation of categorical data in addicted groups, the McNemar test was applied. Paired t test was used to evaluate the data obtained by measuring in dependent groups. The level of statistical significance was accepted as *p* < 0.05.

## Results

The demographic information of the participants and their working status in the COVID-19 related units are summarized in Table 1. A total of 740 healthcare professionals filled out the questionnaire. 66% of population were females (*n* = 495) and 33% were males (*n* = 247). The average age of the population was 35.13 ± 8.35 years.12.4% of the participants are high school graduates, 60.9% are bachelor and 26.7% are masters or above. In addition, 70% are married. Of the participants, 50.9% of the respondents are nurses, 21.4% are medical doctors, 8% are health officer, 6.1% are medical technician, 7.3% are medical secretary and 6.3% are others. At the time the survey was completed, 49.1% of the participants were working in the COVID-19-related units, 44.8% were working in COVID-19 unrelated units and 6.1% were working in both of them.

**Table 1:** Demographic information of the participants

Variables	*n* (%)
Age (mean ± SD) (*n* = 727)	35.13 ± 8.35
Sex (*n* = 742)	
Female	495 (66.3)
Male	247 (33.3)
Education (*n* = 736)	
High school	91 (12.4)
Bachelor	448 (60.9)
Master or above	197 (26.7)
Marital status (*n* = 737)	
Married	521 (70.2)
Unmarried	216 (29.1)
Job title (*n* = 740)	
Nurse	377 (50.9)
Medical doctor	158 (21.4)
Health officer	59 (8.0)
Medical technician	45 (6.1)
Medical secretary	54 (7.3)
Others	47 (6.3)
Epidemic workplace (*n* = 737)	
Covid-19 related unit	362 (49.1)
Covid-19 unrelated unit	330 (44.8)
Both of them	45 (6.1)
Chronic illness (*n* = 740)	
Yes	612 (82.7)
No	128 (17.3)
Type of hospital (*n* = 737)	
Secondary	430 (58.3)
Tertiary	307 (41.7)

The changes in sleep parameters of the study participants before and after the COVID-19 pandemic are summarized in Table 2. Subjective sleep quality deteriorated significantly during the COVID-19 pandemic (poor sleep, 16.9% before COVID-19 versus 52.8% post COVID-19). Sleeping behavior after 24:00 has increased after the COVID-19 pandemic (19.9% before COVID-19 versus 56.1% post COVID-19). In addition, during the COVID-19 pandemic, the duration of the participants' falling asleep was prolonged (falling asleep: 41.75 ± 35.35 min) and the total sleep time was shortened (total sleep time: 6.67 ± 1.88 h). Therefore, the need to take sleeping pills has increased (sleeping pill using: 5.7%). All these results were found to be statistically significant. And also, subjective sleep quality of healthcare professionals working actively during the pandemic was found to be poor (Table 3).

**Table 2:** Sleep parameters of healthcare workers before and after the beginning of COVID-19 pandemic

Variables	Pre COVID-19	COVID-19	*p* value
Subjective sleep quality			
Poor	125 (16.9)	390 (52.8)	0.0001*
Intermediate	165 (22.3)	209 (28.3)	
High	449 (60.8)	140 (18.9)	
Sleep time			
< 24:00	581 (80.1)	316 (43.9)	0.0001*
≥ 24:00	144 (19.9)	404 (56.1)	
Falling asleep (minute)	21.99 ± 17.33	41.75 ± 35.35	0.0001**
Total sleep time (hour)	7.25 ± 1.27	6.67 ± 1.88	0.0001**
Drug using (sleeping pill)	20 (2.7)	42 (5,7)	0.001*

*McNemar test

**Paired *t* test

**Table 3:** Sleep parameters of healthcare workers those working in COVID-19 units and not working in COVID-19 units

Variables	Working in COVID-19 units	Not working in COVID-19 units	*p* value
Subjective sleep quality			
Poor	208 (57.6)	152 (46.2)	0.011*
Intermediate	89 (24.7)	106 (32.2)	
High	64 (17.7)	71 (21.6)	
Sleep time			
< 24:00	131 (37.5)	165 (51.2)	0.001*
≥ 24:00	218 (62.5)	404 (48.8)	
Falling asleep (min)	43.36 ± 38.45	40.45 ± 32.42	0.288**
Total sleep time (h)	6.59 ± 1.86	6.79 ± 1.92	0.171**
Drug using (sleeping pill)	16 (4.4)	18 (5.5)	0.325*

*Chi-square test

**Student *t* test

In the results obtained from the JSS-TR, which is used to evaluate sleep quality, it was determined that sleep quality was statistically significantly impaired in the COVID-19 pandemic (JSS-TR; 4.31 ± 4.53 points before COVID-19 versus 7.52 ± 5.53 points post COVID-19). Furthermore, it has been observed that daytime sleepiness increased after the pandemic (ESS score: 6.08 ± 4.70 in post COVID-19) (Table 4). Those with ESS > 10 before COVID-19 were 3.9%, post-COVID-19 ESS > 10 14.1% (*p* < 0.001). There was no statistical difference in JSS-TR (7.77 ± 5.54 versus 7.07 ± 5.53) and ESS (6.24 ± 4.63 versus 5.71 ± 4.80) values between health workers who were actively working and not working during the pandemic.

**Table 4:** JSS-TR and ESS scores before and after COVID-19

	Pre COVID-19	COVID-19	*p* value*
JSS-TR	4.31 ± 4.53	7.52 ± 5.53	0.0001
ESS	5.36 ± 3.46	6.08 ± 4.70	0.001

*Paired *t* test

## Discussion

There are several studies evaluating the burden of mental health issues (stress, anxiety and depression) and sleep disturbance among healthcare providers during COVID-19. The prevalence of sleep problems is high and affects approximately 40% of people from general and health care populations. No systematic review or meta-analysis has yet been conducted to examine the impact of the pandemic on the prevalence of sleep problems among the general population, healthcare professionals, or COVID-19 patients [1–3]. A recent systematic review evaluated the impact and prevalence of sleep problems among the general population, healthcare workers, or COVID-19 patients of the pandemic. They found that health care workers and the general population had comparative rates of sleep problems with rates of 36.0% (95% CI 21.1–54.2%) and 32.3% (95% CI 25.3–40.2%), respectively [11]. The prevalence and severity of sleep problems in different populations remains unknown. We evaluated how sleep behaviors changed in a large population of healthcare workers during the COVID-19 pandemic in Turkey, the presence of excessive daytime sleepiness, and their sleep quality with the JSS, which is an easily applicable scale.

Healthcare workers who participated in our survey reported that they started to go to bed later, fell asleep later (mean: 41.75 ± 35.35 min), their total sleep time (mean: 6.67 ± 1.88 h) was shortened, and they needed medication to sleep more (5.7%) after the COVID-19 pandemic. During the COVID-19 pandemic, bedtime behavior after 24:00 decreased from 80.1 to 43.9% of those who previously went to bed before 24:00. For those who went to bed after 24:00 before, it increased from 19.9 to 56.1%. In addition, sleep quality as assessed by subjective and JSS significantly deteriorated after the COVID-19 pandemic. Excessive daytime sleepiness increased. Those with ESS > 10 before COVID-19 were 3.9%, post-COVID-19 ESS > 10 14.1% (*p* < 0.001).

Mental health status of healthcare workers worldwide during the COVID-19 pandemic; It has been shown to be affected by high levels of psychiatric symptoms, including anxiety, depression, acute stress, PTDS, and sleep disturbances. Sleep problems may be associated with other disorders, such as: PTSD, depression, anxiety. Two factors can contribute to sleep problems among healthcare workers: high workload (including night work that alters circadian rhythms) and stress-induced sleep problems [1, 12, 13]. Global prevalence reports of 20–45% for insomnia symptoms during the COVID-19 pandemic [3]. Sleep deprivation leads to cognitive impairment and reduces cognitive processing affecting everything from memory to reflexes. This is an important risk factor for health workers to make wrong decisions and for important mistakes and work accidents. Moreover, inadequate and poor sleep affects the immune system and mental health, impairs the immune response, facilitates the spread of infectious diseases, and worsens mental health and quality of life [1, 11, 14]. The sleep behaviors and sleep quality of our participants were adversely affected by the COVID-19 pandemic. The deterioration in sleep quality was independent of active work in the pandemic. This suggested that even if all healthcare professionals do not care for COVID-19 patients, working in a high-risk area may be sufficient to disrupt their sleep patterns.

The Pittsburgh Sleep Quality Index (PSQI) captures a very broad range of sleep-related issues (e.g., nightmares, snoring, sleep medication use), which may explain the higher prevalence rates compared with the Athens Insomnia Scale, Insomnia Severity Index, or researcher developed measure. Findings on sleep problems were obtained using the PSQI, suggesting that health care providers and the general population were affected comparatively with rates of 39.7% (95% CI 21.2–61.6%) and 37.9% (95% CI 25.2–52.4%), respectively [11]. We preferred JSS to assess sleep quality, as PSQI is more difficult to applicability and computation in large population studies. We also asked about the subjective sleep quality as poor, moderate, high. In the COVID 19 pandemic, very few studies have used the JSS [15, 16] and there is no study conducted in healthcare workers. It is very significant and practical to use in large population studies to evaluate the effects of the pandemic on sleep quality.

Also, ESS is often used in large studies. We found that daytime sleepiness increased during the pandemic in healthcare workers who participated in our study. Healthcare workers are particularly at risk of sleepiness affecting their jobs as they tend to work long shifts or work at night. This was accompanied by depression, anxiety and working under stress. Excessive sleepiness and fatigue can lead to deterioration in cognitive functions, leading to serious errors and accidents in the workplace [17–20]. Therefore, training on sleep hygiene and coping with fatigue should be given to healthcare professionals. It is necessary to try to control various behavioral and environmental factors that may adversely affect sleep quality and duration [21, 22].

## Conclusions

The COVID-19 pandemic has significantly adversely affected the sleep behavior and sleep quality of healthcare professionals. The JSS is an easily applicable scale for assessing sleep quality in large population studies. Prevention and therapeutic strategies of sleep disorders during COVID-19 and the pandemic will have a crucial contribution to healthcare workers making the best decisions when applying established treatment guidelines, protecting their physical and mental health, and even strengthening their immune response to illness.

### Study limitations

Our study was conducted in 2 pandemic hospitals in the city center, and it was one of our limitations that it did not include health care workers working in rural areas and primary care centers. Another limitation was that the survey could not be applied face to face due to pandemic, and the data were collected through responses to online survey questions. Finally, since we do not have any recorded data about sleep characteristics of healthcare workers before COVID-19 era, we evaluated sleep characteristics of the participants prior to COVID-19 pandemic based on their statements. Therefore, the memory factor may have a role in the questioning of sleep characteristics of participants prior to COVID-19 Pandemic.

## Data Availability

The data sets generated and/or analysed during the current study are not publicly available due but are available from the corresponding author on reasonable request.

## Abbreviations

COVID-19: Coronavirus disease-19
SARS COV-2: Severe acute respiratory syndrome coronavirus 2
PTSD: Post-traumatic stress disorder
JSS: Jenkins Sleep Scale
JSS-TR: Jenkins Sleep Scale Turkish version
ESS: Epworth Sleepiness Scale
PSQI: Pittsburgh Sleep Quality Index

## References

[1] Lazzari C, Shoka A, Nusair A, Rabottini M (2020). Psychiatry in time of COVID-19 pandemic. Psychiatr Danub.

[2] Al Maqbali M, Al Sinani M, Al-Lenjawi B (2021). Prevalence of stress, depression, anxiety and sleep disturbance among nurses during the COVID-19 pandemic: a systematic review and meta-analysis. J Psychosom Res.

[3] Pappa S, Ntella V, Giannakas T, Giannakoulis VG, Papoutsi E, Katsaounou P (2020). Prevalence of depression, anxiety, and insomnia among healthcare workers during the COVID-19 pandemic: a systematic review and meta-analysis. Brain Behav Immun.

[4] Silva ESME, Ono BHVS, Souza JC (1992). Sleep and immunity in times of COVID-19. Rev Assoc Med Bras.

[5] Tu ZH, He JW, Zhou N (2020). Sleep quality and mood symptoms in conscripted frontline nurse in Wuhan, China during COVID-19 outbreak: a cross-sectional study. Medicine (Baltimore).

[6] Shreffler J, Petrey J, Huecker M (2020). The impact of COVID-19 on healthcare worker wellness: a scoping review. West J Emerg Med.

[7] Jenkins CD, Stanton BA, Niemcryk SJ, Rose RM (1988). A scale for the estimation of sleep problems in clinical research. J Clin Epidemiol.

[8] Duruöz MT, Erdem D, Gencer K, Ulutatar F, Baklacıoğlu HS (2018). Validity and reliability of the Turkish version of the Jenkins Sleep Scale in psoriatic arthritis. Rheumatol Int.

[9] Johns MW (1991). A new method for measuring daytime sleepiness: the Epworth Sleepiness Scale. Sleep.

[10] Izci B, Ardic S, Firat H, Sahin A, Altinors M, Karacan I (2008). Reliability and validity studies of the Turkish version of the Epworth Sleepiness Scale. Sleep Breath.

[11] Jahrami H, BaHammam AS, Bragazzi NL, Saif Z, Faris M, Vitiello MV (2021). Sleep problems during the COVID-19 pandemic by population: a systematic review and meta-analysis. J Clin Sleep Med.

[12] Marvaldi M, Mallet J, Dubertret C, Moro MR, Guessoum SB (2021). Anxiety, depression, trauma-related, and sleep disorders among healthcare workers during the COVID-19 pandemic: a systematic review and meta-analysis. Neurosci Biobehav Rev.

[13] Korkmaz S, Kazgan A, Çekiç S, Sağmak Tartar A, Balcı HN, Atmaca M (2020). The anxiety levels, quality of sleep and life and problem-solving skills in healthcare workers employed in COVID-19 services. J Clin Neurosci.

[14] Kim-Godwin YS, Lee MH, Logan JG, Liu X (2021). Factors influencing sleep quality among female staff nurses during the early COVID-19 pandemic in the United States. Int J Environ Res Public Health.

[15] Pinto J, van Zeller M, Amorim P, Pimentel A, Dantas P, Eusébio E (2020). Sleep quality in times of Covid-19 pandemic. Sleep Med.

[16] ToprakCelenay S, Karaaslan Y, Mete O, Ozer KD (2020). Coronaphobia, musculoskeletal pain, and sleep quality in stay-at home and continued-working persons during the 3-month Covid-19 pandemic lockdown in Turkey. Chronobiol Int.

[17] Uehli K, Mehta AJ, Miedinger D, Hug K, Schindler C, Holsboer-Trachsler E (2014). Sleep problems and work injuries: a systematic review and meta-analysis. Sleep Med Rev.

[18] Epstein M, Söderström M, Jirwe M, Tucker P, Dahlgren A (2020). Sleep and fatigue in newly graduated nurses-experiences and strategies for handling shiftwork. J Clin Nurs.

[19] Lo WY, Chiou ST, Huang N, Chien LY (2016). Long work hours and chronic insomnia are associated with needlestick and sharps injuries among hospital nurses in Taiwan: A National Survey. Int J Nurs Stud.

[20] Abbas A, Al-Otaibi T, Gheith OA, Nagib OA, Farid MM, Walaa M (2021). Sleep quality among healthcare workers during the COVID-19 pandemic and its impact on medical errors: Kuwait experience. Turk Thorac J.

[21] Xiao H, Zhang Y, Kong D, Li S, Yang N (2020). The effects of social support on sleep quality of medical staff treating patients with coronavirus disease 2019 (COVID-19) in January and February 2020 in China. Med Sci Monit.

[22] Becker PM (2022). Overview of sleep management during COVID-19. Sleep Med.

